# Mycelium vs. Fruiting Bodies of Edible Fungi—A Comparison of Metabolites

**DOI:** 10.3390/microorganisms10071379

**Published:** 2022-07-08

**Authors:** Ralf G. Berger, Sven Bordewick, Nina-Katharina Krahe, Franziska Ersoy

**Affiliations:** Institute of Food Chemistry, Leibniz University Hannover, Callinstraße 5, 30167 Hannover, Germany; rg.berger@lci.uni-hannover.de (R.G.B.); sven.bordewick@lci.uni-hannover.de (S.B.); nina.krahe@lci.uni-hannover.de (N.-K.K.)

**Keywords:** basidiomycota, *Agaricus bisporus*, *Pleurotus ostreatus*, *Lentinula edodes*, edible fungi, fermentation, mycelium production, fruiting bodies, chemical composition

## Abstract

Edible mushrooms are widely appreciated for their appealing flavours, low caloric values and high content of presumably health-protecting metabolites. Their long history of safe use together with the looming worldwide food crisis have revived the idea of generating meat analogues and protein isolates by the controlled fermentation of mycelia of these edible fungi as a dietary option. The occurrence of proteins, polysaccharides, smaller metabolites, metal ions and toxins in mycelia and fruiting bodies is compared among the three most popular species, *Agaricus bisporus* (button mushroom), *Pleurotus ostreatus* (oyster mushroom), *Lentinus edodes* (shiitake) and some closely related species. Large effects of substrate chemistry, strain, developmental stage and ecological interactions result in a wide variation of the concentrations of some metabolites in both mycelial cells and fruiting bodies. This is obviously a result of the high adaptation abilities required to survive in natural habitats. Fungal bioprocesses are decoupled from agricultural production and can be operated anytime, anywhere, and on any scale according to demand. It is concluded that fungal biomass, if produced under food-grade conditions and on an industrial scale, could provide a safe and nutritious meat substitute and protein isolates with a high biological value for future vegan foods.

## 1. Introduction

The millennium goals of the United Nations, the program *Horizon Europe* 2021/22 and the *Green Deal* of the European Commission define the creation and safeguarding of a sufficient and nutritious diet for the growing population of the world as one of the “*Grand Challenges*” [1,2,3]. In a technical sense, all food sources (plants, animals, microorganisms) are “sustainable”, but in a more integrated sense, all economic, ecological and social dimensions of food production and distribution should be considered. Current food production accounts for more than one third of all greenhouse gas emissions, cultivation of cattle and rice being the largest contributors with 25 and 12%, respectively [4]. Conversely, climate change threatens food production through droughts, storms, soil degeneration and the loss of biodiversity. A disruptive change to the current status of the food industry towards more resource-saving and yield-optimised production is essential, but how such change can be achieved remains an open question. 

The shortest remedy was formulated by a famous musician: “*Less meat—less heat*” [5]. Alternative protein sources derived from legumes (soy, peas, beans, etc.), cereals and nuts have appeared in marketed meat substitutes. The same applies to proteins from some algae and cyanobacteria (*Chlorella* and *Spirulina*), insects (for example from *Alphitobius diaperinus*, the lesser mealworm) and moulds (*Quorn* from *Fusarium venenatum*). However, cultivation of plants requires arable land, a stable environment with sufficient rain, pesticides and harvesting machinery. In addition, harvesting is possible only once per season. Algae turn black when heated or extruded [6], insects suffer from the neophobia problem and cultural concerns, and moulds constantly receive a bad press. The development of chicken and bovine protein production using heterologous hosts (“precision fermentation”) and the 3D-printing of meat substitutes from differentiated animal stem cells (“cultured meat”, “clean meat”) are still in their infancy.

**Figure 1:**
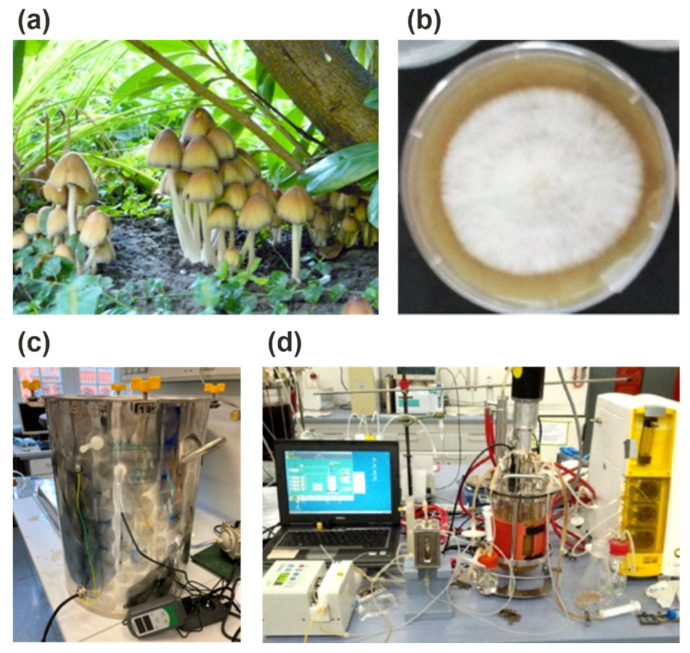
(**a**) The typical morphology of a xylophilic basidiomycete with stipe and pileus growing on the rotting roots of a willow tree (here: *Coprinus micaceus*). (**b**) Emerse cultivation of *Pleurotus sapidus* on agar plates supplemented with 5% (*w*/*v*) lemon peel. (**c**) Simple temperature controlled and aerated stainless-steel tank for the solid-state cultivation of mycelium of a basidiomycete. (**d**) Submerged cultivation in a lab-scale fermenter requires more complex peripheral instrumentation, such as pH and oxygen probes, pumps and electronic data processing (© pictures by the authors).

In this context, it is surprising that the long-standing idea to propagate the mycelia of edible fungi on a fermenter scale [7] has not been pursued more intensively. Edible fungi (“mushrooms”, Basidiomycota) possess attractive nutritive, sensory, and presumed health-promoting properties [8]. They have a long history of safe use, and may be grown without the need for much space or energy, using various fermenter types in a controlled environment and using inexpensive lignocellulosic side-streams of the agro-industrial production of foods, such as pomace, brans, fibres and peels, as substrates [9] (Figure 1).

The central question to be answered by the following comparison of the chemical constituents of fermenter grown mycelia and fruiting bodies of edible fungi is whether such mycelia and the protein-rich fractions derived from them can be regarded as safe and used as food or food ingredients, if produced in a food-grade environment. For the sake of brevity, this text will restrict itself to recent work on the three best investigated and most popular species: *Agaricus bisporus* (J. E. Lange) Imbach, *Pleurotus ostreatus* (Jacq.: Fr.) Kumm., and *Lentinus edodes* (Berk.) Singer (shiitake) [10].

## 2. From Mycelium to Fruiting Body: Differential Gene Expression

The shape of a typical epigeal fruiting body with stipe, pileus and gills (Figure 1a) differs visibly from hypogeally growing mycelia, the cotton-like cell cultures on agar plates (Figure 1b) or the cell clusters produced in a fixed bed or stirred tank fermenter (Figure 1c,d). These cells are exposed to different conditions of relative humidity, partial pressure of oxygen, light, temperature, shear stress and nutrients. The morphological differences result from differential gene expression, and not from alterations of the genome. The transition from parallelized dikaryotic mycelial cells to primordia to the developing pseudotissue of a fruiting body is fluent. Genetic changes occur only during meiosis and spore formation in the basidia (see below). An immediate conclusion would be that genomes lacking the information for the formation of toxins or other adverse compounds (for example polyketide synthase genes) will always form toxin free mycelia and fruiting bodies. Substantial chemical equivalence (not identity) exists on the genetic level between parental dikaryon and daughter monokarya.

Recent studies using high-throughput techniques have shed light on the differential gene expression during fruiting body formation in *L. edodes*. An Illumina HiSeq based study counted 2080 differentially expressed genes. 1503 were upregulated in the mycelium, and 577 in a mature fruiting body. The fruiting body-specific transcripts were related to replication, karyogamy, and meiosis during fruiting body maturation [11]. Differential gene expression does not stop during growth and aging of the fruiting body. Changes were followed from the early bud to the intermediate developing stage to the fully developed stage; highly active genes were related to purine and unsaturated fatty acid metabolism and, as expected, to meiosis [12]. Lectins (non-immunoglobulin carbohydrate binding proteins) were also shown to occur specifically in the vegetative mycelium, fruiting body or primordium [13]. In addition, the fungi’s genetic background for the formation of volatile flavour compounds was evaluated by an expression study, which identified two optimal reference genes for both the mycelium and the fruiting body [14].

A recent study on *Agaricus blazei* (now *Agaricus subrufescens* Peck), an edible species closely related to *A. bisporus*, yielded more than 10,000 putative genes, and many of them were differentially expressed in mycelia, primordia and fruiting bodies [15]. The latter showed a higher activity of genes associated with stress response, ribosome biogenesis, arginine biosynthesis, and steroid biosynthesis [15]. A follow-up study identified *μ*mol/L concentrations of methyl jasmonate and salicylic acid as potent inducers of ergosterol synthesis in liquid cell cultures. Within a short incubation, the concentrations of ergosterol in the mycelium exceeded those in the fruiting body [16]. In contrast, genes coding for hydrophobin formation in *A. bisporus* were preferentially expressed in the peel of the fruiting body, which agrees with their presumed role as a protective hydrophobic layer on the pileus surface [17].

The bipolar hydrophobin proteins tend to self-assemble in monolayers, which explains both their physiological role as a coating and their possible technological function as an emulsifying agent. They can serve as a guideline for gene expression studies in different stages of the fungal life cycle. Of the 40 hydrophobin genes that were differently expressed temporally and spatially in *P. ostreatus*, some were abundant in mono- and dikaryotic mycelia, others were expressed only in mono- or dikaryotic mycelia, and others again were expressed preferably in fruiting bodies. The substrate, natural light and temperature affected hydrophobin gene expression and the subsequent growth rate and primordia formation [18]. The influence of light on fruiting body formation has been supported by the identification of two blue light receptor genes in *P. ostreatus* [19].

A recent transcriptome analysis is also available for *Pleurotus eryngii* (kings trumpet), a close relative of *P. ostreatus* [20]. A comparison of transcriptomics of mature mycelia and primordia showed a total of 10,718 upregulated and 8937 downregulated genes in the primordium library. The differentially expressed genes were associated with cell wall degradation, carbohydrate hydrolysis, light perception, and cAMP signal transduction. The dramatic changes explain the observed morphological changes during fruiting body formation.

## 3. From Genomics to Proteomics: Proteins, Enzymes and Amino Acids

If fermenter produced mycelia were to be introduced as alternative protein sources, a high protein concentration would be desired. Depending on the literature source, cultivation conditions and strain, the total protein concentration of fruiting bodies of the *Agaricus* species ranged from 24 to 48% in dry matter, for *Pleurotus* species from 15 to 33% and in *Lentinula* from 10 to 20% [21,22,23,24,25,26]. The few sources directly comparing protein concentration in fruiting body and mycelium found similar values [22,27]. These protein levels compete favourably with those of leguminous plants, such as pea, chickpea, soy or lupine [18]. In the fermentation processes, the mycelia are suspended in a liquid nutrient broth. Assuming a final biomass yield of 100 g/L and a water content of the mycelia of 90%, a proximate protein yield of 3 g/L can be estimated, which is consistent with experimental data [22].

Protein isolates can be obtained from disintegrated mycelia by common combinations of extraction, (isoelectric) precipitation and centrifugation. *Quorn*, a food preparation containing protein isolated from an ascomycete, was introduced on the UK food market in the early 1980s and no adverse effects have been reported since [28]. Literature on mushroom allergies is very scarce, indicating a generally very low incidence from human consumption. Woodland basidiomycetes produce large quantities of aerospores, in temperate zones mainly in August and September, and inhalation is inevitable, but even here hard clinical data is missing. The risk of an abnormal immune response is further decreased by the fact that the preparation of a meat protein substitute will always have to include a thermal denaturing step.

A complex metabolism driven by enzymes lies beneath the protein concentrations summarised above. In *A. bisporus*, laccase concentration increased during mycelial growth and declined at the onset of fruiting body formation, while cellulase activity increased at fruiting. No such changes were observed for most other enzymes [29]. The expression of genes coding for zinc finger proteins in *P. ostreatus* was regulated by developmental processes, phytoeffectors and abiotic stress [30], whereas, in line with a genetic study [13], the spectrum of lectins changed from mycelium to primordium to mature fruiting body [31]. A lentinan degrading *β*-1,3-glucanase of *L. edodes* occurred after the harvest of fruiting bodies but was not detectable in mycelia and young fruiting bodies [32].

All essential amino acids were present in mycelial proteins, but similar to the protein fraction, absolute numbers deviate from source to source. Glutamic acid was highest in *A. bisporus* [27], *P. sapidus* and *P. sajor-caju* [22], whereas aspartic and glutamic acid dominated in *Lentinula* mycelium [22]. When the biological value (BV) was calculated according to the FAO/WHO reference, the mycelial proteins ranged from BV 66 (*L. edodes*) to BV 82 (*Pleurotus* sp.), thus equalling or even exceeding common plant sources, such as bean (BV 58) or even soy, the most valuable plant protein (BV 74) [22]. Among *Pleurotus* fruiting bodies cultivated on mixed wheat straw and sugar beet compost, a large inter-species and even inter-strain variation in protein concentration (calculated as N × 4.38) from 22.4 to 34.7% and glutamate (calculated as percentage of total amino acids) from 12.8 to 18.7% was observed. *L. edodes* contained only 15.2% protein, but the highest glutamate concentration with 20.9% [33]. The data explain why mushrooms are popular food ingredients and side dishes: Glutamic and, to a lesser extent, aspartic acid (and certain peptides containing them), are taste enhancers and used in many salty/sour convenience foods such as soups and sauces [34,35].

## 4. Polysaccharides

Since ancient times, the consumption of edible fungi has been linked with health claims, such as preventing Parkinson’s and Alzheimer’s disease, diabetes, hypertension, stroke as well as antitumor, antibacterial, immune boosting and cholesterol lowering properties [8]. Many of these presumed benefits were linked to the occurrence of various branched polysaccharides. Extending earlier work [36], a recent study on polysaccharides from *A. bisporus* fruiting bodies, cultured mycelia, and fermentation broth showed that the polysaccharides of the fruiting body were mainly composed of mannose, whereas the mycelia contained mainly glucose and the cultivation medium mainly galactose [37]. Dynamic changes in yield and the molecular mass of polysaccharides consisting of fructose, galactose, glucose and mannose were found during the developmental stages of fruiting bodies of *L. edodes* [38]. A recent review summarised the work on these polysaccharides including the isolation methods, structural features, bioactivity mechanisms and structure-activity relationships [39]. Focussing on tumour-suppressing properties (among others lymphocyte proliferation and macrophage activation) in a mouse model, glycans isolated from the mycelia and fruiting body of *P. ostreatus* showed very similar properties as “immunoceuticals” [40]. To produce polysaccharide preparations in sterile submerged cultures would provide numerous advantages over the laborious manual harvest of fruiting bodies. In line with this, work on the closely related edible *P. eryngii* concluded “that the polysaccharides from mycelia can be an effective substitute of polysaccharides from fruiting body” [41].

## 5. Small Metabolites

**Figure 2:**
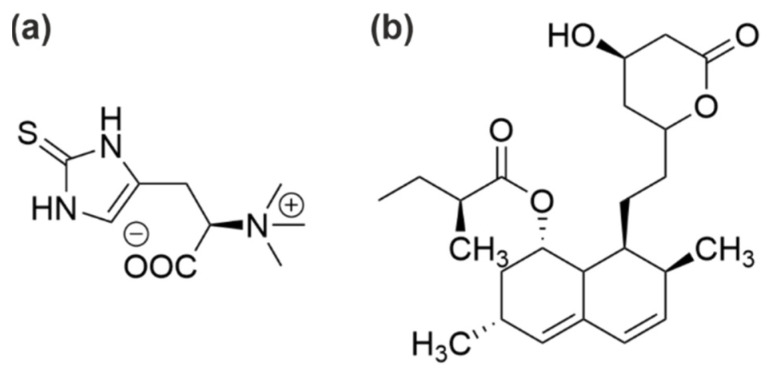
(**a**) Ergothioneine is a rare thiourea derivative of histidine, named after the ergot fungus *Claviceps* and sold as dietary supplement, although its benefit to human metabolism is uncertain [43]. (**b**) Lovastatin is a cholesterol lowering polyketide and a genuine constituent of edible mushrooms. It inhibits 3-Hydroxy-3-methylglutaryl-CoA reductase [44].

In a search for compounds with antioxidant and cytotoxic capacities, mycelia of *A. bisporus* and *P. ostreatus* were cultured in different solid and liquid media [42]. *P. ostreatus* mycelia accumulated higher concentrations of ergosterol and phenolic compounds than the corresponding fruiting body and showed cytotoxicity toward tumour cell lines. In contrast, fruiting bodies of *A. bisporus*, when compared to young and old mycelia, contained higher concentrations of antioxidant phenols and ergothioneine (Figure 2a) [42,43].

A comparison of the production of volatile compounds by liquid, surface, and solid support cultures with the fruiting body of *P. ostreatus* by dynamic headspace sampling and coupled gas chromatography-mass spectrometry and gas chromatography-olfactometry showed that aroma compounds with the typical mushroom odour, such as octan-3-one and octan-3-ol, were present in the same proportions in mycelia from surface and solid support cultures and in the fruiting body, but not in submerged grown mycelia [45]. This is a promising finding, because it allows the easy incorporation of fungal biomass and extracted proteins produced under submerged conditions into sweet or sour/salty foods without affecting their sensory profiles. The analysis of the fatty acid composition of fruiting body *vs*. mycelium supported this, as the presumed precursor of the eight carbon volatiles, linoleic acid, occurred in much lower concentration in submerged grown mycelium than in fruiting bodies (16 vs. 55.5%) [46]. When cultivated in a xylose/corn steep liquor medium, the mycelium contained more saturated and mono-unsaturated fatty acids, while the ω6/ω3-ratio within the fraction of polyunsaturated fatty acids was nearly identical.

Mycelia of *L. edodes* contained a low concentration of mannitol (ca. 1% in dry mass) whereas the fruiting body contained 20 to 30% in stripe and pileus, respectively, which was exceeded by the pileus of *A. bisporus* (almost 50%). The most likely explanation for the high mannitol concentration is the need to maintain the osmotic pressure of the soft fruiting bodies until the onset of sporulation [47]. Such sugar alcohols are approved food additives in the European Community (mannitol is E421), but act as laxatives and diuretics in higher dosages [48], which would favour the consumption of mycelial preparations, particularly when considering the presence of higher levels of non-digestible and intestine motility accelerating polysaccharides (see section Polysaccharides).

Like many other edible fungi, the fruiting bodies of *A. bisporus* and *L. edodes* are a source of lovastatin, a cholesterol-lowering compound (Figure 2b). A comparative study found the lowest concentration in the fruiting bodies of *L. edodes* (around 1 mg/100 g dry mass). However, the amount of lovastatin released from the extracts of the examined species into digestive juices was highest for the mycelium from in vitro cultures of *L. edodes* (0.51 mg/100 g dry mass). It was concluded that *L. edodes* mycelium represented “a product with increased hypolipidemic activity” [44].

## 6. Minerals

The accumulation of heavy metals including radionuclides in fruiting bodies is well-known. Comparing young and old mycelia and fruiting bodies of *A. bisporus*, the lowest concentrations of Ni, Pb, Cd, and Cr were found in mycelium samples. As expected, rather high concentrations of Cu, Zn, Fe and Mn were found in fruiting bodies [43]. Accumulation in fruiting bodies depends on the presence of bioaccessible metal fractions in the soil. Correlations between concentrations in soil and fruiting bodies were established for essential micronutrients, such as Cu, Mn, Zn and Ni, but only the levels of Pb in fruiting bodies were close to toxicologically alarming levels [49].

Observations of this kind stimulated the idea to supply growing mycelia or fruiting bodies with metal salt solutions during growth to complement possible deficiencies in human nutrition (“biofortification”). An extensive study measured similar concentrations of Mg ions in mycelium and fruiting body of *A. bisporus*. Addition of Zn ions to in vitro cultures increased Mg uptake, while addition of l-tryptophan stimulated the uptake of Zn and Fe [50]. Cd concentrations were much lower in mycelia than in fruiting bodies. Generally, large biological deviations of the metal ion concentrations were recorded in samples of fruiting bodies. Fe concentrations in fruiting bodies of *A. bisporus* ranged from about 14 to 160 μg/g dry mass, and the l-tryptophan enriched in vitro cultures reached about 119 to 312 μg Fe/g dry mass [50]. Similar data were provided from in vitro cultures of *P. eryngii* [51]. Its ability to accumulate Zn and Se from the culture medium depended on the type of salt used as a supplement. Enrichment of Cu, Zn and Se was also demonstrated for the mycelium of *L. edodes*, while much lower concentrations were reached in fruiting bodies [52]. Thus, in vitro cultured mycelium can be grown in chemically defined, mineral-enriched nutrient media resulting in tailored food supplements with known and safe mineral concentrations.

The concept also worked with supplemented Lithium salts [53]. Lithium therapy is used to treat bipolar disorders and neurodegenerative brain diseases, but side effects were reported. Mycelia of both *A. bisporus* and *P. ostreatus* were able to accumulate Li and were suggested “as an alternative, more calibrated and bio-accessible source” [53]. Enrichment levels up to 400-fold relative to retail mushrooms were measured, resulting in enormous 1.6 g Li/kg dry mass in the mycelia of *P. ostreatus*. The authors assumed that the longer period of digestion and uptake would result in more favourable pharmacokinetic properties for the Li ions and thus reduce the side effects. The mycelia of these fungi appear to contain efficient metal ion transporters. Thus, the necessary concentrations of metal ions have to be carefully assessed in order not to produce excessively high concentrations.

## 7. Effects of the Chemical Environment on the Concentration of Metabolites

The strong dependence of growth and metabolite formation on the chemical surroundings reflects the high potential of fungi to adapt to different and changing environmental conditions. This ability allows the use of agro-industrial side-streams as growth substrates of submerged cultivated mycelia. The side-streams should ideally satisfy certain criteria. They should be of food origin, low priced, high volume, locally produced, rich in nutrients, compatible with the physiology of the fungus, free from growth inhibitors, and easy to mill and sterilise (Table 1).

The almond mushroom *Agaricus subrufescens* (Syn. *A. brasiliensis*, *A. blazei*), a close relative of *A. bisporus*, was successfully cultivated on straw from asparagus, maize, bean and cotton, and on cottonseed hulls, corn cob sawdust and woodchips, and significant differences in yield, polysaccharide and antioxidant concentrations were found [55,67].

Three strains of *P. ostreatus* were cultivated on sunflower husks and barley straw supplemented with corn husks, wheat bran, rye malt, and soy flour [57]. The supplementation resulted in distinct shifts in the composition of volatile compounds in the fruiting bodies. Apart from mushroom notes, herbaceous, floral, and sweet aroma compounds were also formed. Obviously, the growth substrates govern, to some extent, the sensory and perhaps also the medicinal attributes of the fruiting bodies, as was suggested by the authors. Numerous in vitro cultivated species of the genus *Pleurotus* and the emerging fruiting bodies were compared and analysed for presumed health-promoting constituents [69]. Fruiting bodies contained high concentrations of phenolic and indole compounds, whereas the mycelia obtained from in vitro culture contained more bioactives such as lovastatin (Figure 2b).

Investigating lignolytic enzymes, large differences in the activities of laccase, horseradish-like- and Mn-peroxidase and superoxide dismutase were determined in the mycelia, primordia and fruiting bodies of *P. ostreatus* and *P. sajor-caju* [70]. For example, a high manganese peroxidase activity was detected only in fruiting bodies of *P. ostreatus*, in contrast to the low activity in *P. sajor-caju*. High activities of the horseradish-like peroxidase and laccase were found in the primordia and a high concentration of methoxyphenols in the mycelia of *P. ostreatus*, which was about 100 times higher compared with *P. sajor-caju*.

Not only aroma attributes [57], but also tastants determine the acceptance of mushrooms by consumers. A comprehensive study on the fruiting bodies of *L. edodes* found pronounced effects of the substrate, and in particular of the C/N-ratio, on the formation of soluble sugars, polyols, organic acids, sweet and essential amino acids, umami 5′-nucleotides, trehalose, arabitol, malic acid, succinic acid, mannitol and citric acid [58]. Comparing six edible fungi, among them *L. edodes* and *P. eryngii*, and two common substrates (alder and beech sawdust or oak sawdust and flax shives, respectively) a high affinity of *L. edodes* to Cd was found, whereas other edible species selectively accumulated Al, Ni, Pb or Hg [71]. Mixed agro-industrial side-streams were also used in a study on enzyme activities (laccase, cellulose, xylanase), yield, firmness and mineral composition of *L. edodes* [56].

Most of the works cited in this chapter were driven by attempts to improve large-scale production of fruiting bodies and showed the immense effects of the chemical environment on the formation of smaller metabolites, accumulation of minerals and activity of enzymes. Comparisons with mycelia were largely missing. However, a study on *Tyromyces floriformis*, a sesquiterpene producing basidiomycete with uncertain palatability, showed dramatic responses of submerged cultures to polysaccharides and lipids in the growth medium [72]. Rye arabinoxylan inhibited sesquiterpene synthesis, but the addition of its building blocks arabinose, xylose, or ferulic acid showed no effect, whereas only 0.05% (*w*/*v*) 3^2^-*α*-l-arabinofuranosyl-xylobiose resulted in a significant suppression. In contrast, the supplementation of the cultivation medium with acetyl donors boosted the sesquiterpene concentration. The effects on sesquiterpene synthesis correlated with the intracellular sesquiterpene cyclase activity. Similar results were obtained using submerged cultures of *Cerrena unicolor*, *Postia placenta*, and *Coprinopsis cinerea*, indicating that the metabolism of mycelia is likewise highly susceptible to the extracellular chemistry.

## 8. Chemical Composition of Mycelia and Fruiting Bodies Differs Depending on Strain

In the complex course of the cell cycle of higher fungi, monokaryotic (haploid) cells fuse to dikaryots, and dikaryots fuse in the basidia of mature fruiting bodies. Plasmogamy and karyogamy generate single diploid cells, which enter meiosis, a process eventually resulting in four new monokarytic spores with slightly different genomes. The new cell lines arising from spore germination differ accordingly in growth rate as a global parameter and also in their biochemical properties. The haploid cells may develop into a new mycelium or fuse, if appropriate mating types meet, to a new diploid cell with two individual nuclei. This diploid state may persist until new fruiting bodies are formed. As a result, a large genetic space is formed in which mono- and dikaryotic cells co-exist and grow worse or better depending on their ability to adapt to the actual habitat [73].

**Figure 3:**
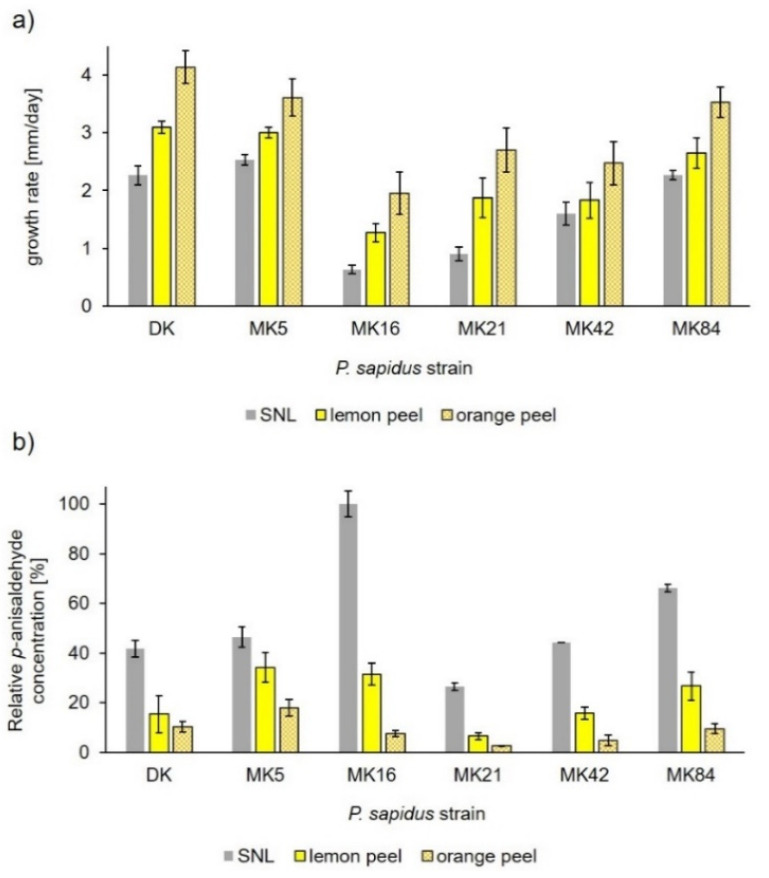
Influence of different cultivation media on the growth rate and alkene cleavage activity of *P. sapidus*. (**a**) Radial growth rate on agar plates. (**b**) Enzymatically generated concentration of *p*-anisaldehyde after cleavage of *trans*-anethole using finely ground lyophilized mycelium. Concentrations were calculated relative to the highest *p*-anisaldehyde concentration. Lemon peel: MM supplemented with 5% lemon peel (*w*/*v*), orange peel: MM supplemented with 5% orange peel. DK—dikaryon, MK—monokarya [60].

For example, one hundred spores were collected from a single fruiting body of *P. sapidus*, a close relative of *P. ostreatus*, and grown submerged in standard nutrient liquid (SNL) or in minimal medium supplemented with lemon or orange peel [60]. Significant differences of the growth rate were found. Some monokaryotic strains grew more slowly or as fast as the parent dikaryon. The supplements had a positive effect on mycelial growth for all strains, which was more pronounced for orange peel (Figure 3a). As mentioned above [57], different strains may be distinguished by their pattern of volatiles. Thus, the cleavage of the alkene side chain of *trans*-anethole to *p*-anisaldehyde was selected as an indicator reaction to evaluate biochemical differences. In contrast to the faster growth of all cultures in the presence of orange and lemon peel, it had a negative effect on the enzymatic activity. Significant differences between monokaryotic strains, and in comparison with the dikaryotic parent strain, were detected for the production of *p*-anisaldehyde (Figure 3b). Thus, the growth rate and enzymatic activity were not correlated, neither for the different strains nor in relation to their response to the growth substrate [60].

Two strains of *A. bisporus* and two of *L. edodes* were grown on liquid static flask cultures and compared for biomass formation, the profile of fatty acids and secondary metabolites [74]. The growth and metabolite production were strain and substrate dependent. Remarkably, expired infant food was used as a medium constituent in these experiments. The investigation of the growth and antioxidant capacity of 27 *Pleurotus* mycelia strains of Malaysian origin identified some “high-quality strains”, whereas others were found inferior [75]. Total phenols, antioxidant capacity and *β*-glucans were found to be diverse in twelve *P. eryngii* isolates from Italy [76]. Very similar results were recently obtained for *L. edodes* by mating monokaryotics and examining agronomic suitability by measuring parameters such as growth rate, pileus pigmentation and size of fruiting bodies [77]. The antioxidant capacity, and tyrosinase and laccase activity were compared for strains of *L. edodes* on the fruiting body level, whilst also looking for a “future genetic breeding program” [78]. 

These findings have inspired the obvious idea to cross the “best” monokarya for a new generation of dikarya with superior properties. However, results were often disappointing, because the selected over-average traits of monokaryotic strains simply did not add up in the new dikarya, a long-standing experience of mushroom growers, but rarely scientifically evaluated and quantified [79]. Our own work generated several hundred monokaryotic strains of *Pleurotus*, which were grouped according to their growth rate on agar plates and compared to the parental dikaryon. The allylic oxidation of the sesquiterpene hydrocarbon (+)-valencene to the grapefruit flavour compound (+)-nootkatone was measured as an indicator reaction [80]. The slowly growing monokarya converted valencene more efficiently to nootkatone. The fast-growing monokaryons and the “slow × slow” and the “fast × fast” dikaryotic crosses showed similar or inferior yields. However, some “slow × fast” dikarya, outperformed the parental dikaryon significantly. Long-term sub-cultivation of the monokaryotic strains showed stable metabolic performance [81]. These results may be attributed to the peculiar physiology of dikaryotic strains: The two nuclei do not fuse but co-exist in the same cell. However, it is difficult to assess whether they communicate with each other or suppress certain traits in a regulated manner, letting one of the two nuclei dominate over the other [82].

## 9. Ecological Interactions Affect Fungal Growth

Many land plant species rely on mycorrhizal relationships with fungi to provide them with inorganic compounds and trace elements, a prime example of mutualistic interactions vital for terrestrial ecosystems. Only recently, mutualistic and also antagonistic interactions were described between *A. bisporus* and related Agaricales and viruses [83], beneficial bacterial contaminants [84,85], synergistic fungi [86] and adverse bacteria and fungi [86,87]. Although the existence of such microbial communities has long been known, only molecular sequencing methods have identified the interacting endogenous or exogenous species. Antagonistic, neutral, and even synergistic interactions among viruses found on fruiting bodies of *A. bisporus* were observed [83]. *Bacillus velezensis* is protective against mould contamination [84], and *Bacillus megaterium* and *Pseudomonas putida* either alone or in combination are effective in improving growth and yield (“biofertilisers”) [85]. Complex consortia consisting of thermophilic proteobacteria and actinobacteria and the thermophilic fungus *Mycothermus thermophilus* were identified [86]. Certain bacteria may protect the pileus against other bacteria [87], while the interaction between asco- and basidiomycetes often finishes with fruiting body decay [88].

A mycovirus impeded growth and formation of extracellular lignolytic enzymes in *P. ostreatus* compared with a virus-cured strain [89], whereas *Glutamicibacter arilaitensis*, a bacterium belonging to the phylum *Actinomycetota* and found on some French cheese, promoted growth and yield [90]. Based on 16 S rRNA gene-terminal restriction fragment length polymorphism, a succession pattern of the microbial community was found, which was thought to protect *P. ostreatus* against competing fungi [91]. Such succession patterns resemble traditional food fermentations, such as sourdough and *Sauerkraut*, where one microorganism paves the way for the next one. It is expected that studies in the near future will shed light on the metabolic changes leading to the improved growth and yield observed for the beneficial “contaminants”.

## 10. Toxins in Edible Fungi

**Figure 4:**
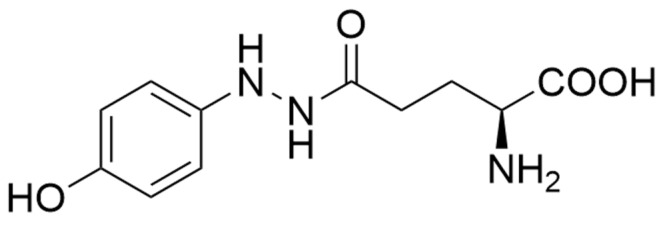
Agaritine, a genuine toxin of *A. bisporus*, is degraded by heating or freezing.

According to the *Bundesinstitut für Risikobewertung* almost every “edible” fruiting body may cause symptoms of intolerance [92], and some even contain true poisons. Cultivated *A. bisporus* may accumulate up to 0.5 g agaritine per kg, a fortunately heat-labile hydrazine and precursor to carcinogenic compounds capable of adding on DNA (Figure 4) [93,94]. The increase in vegetarian diets has led to a higher consumption of raw fruiting bodies. This, in turn, has resulted in higher levels of intoxications [92]. Fruiting bodies of *Agaricus* harvested later in the cropping cycle produced more agaritine than younger samples, while mycelium growing in liquid culture showed much lower levels than present in the fruiting bodies [93].

Ostreolysin A is a toxic cytolysin of *P. ostreatus* that perforates membranes of erythrocytes, thus resulting in a non-selective permeability towards ions and small neutral solutes [95]. Dermatitis can be caused by skin contact with *L. edodes* [96]. As with *A. bisporus*, it is recommended to avoid consumption of raw or undercooked *P. ostreatus* and *L. edodes* fruiting bodies [92]. For the intended use of fungal mycelia or protein extracts as meat substitutes, a thermal processing step will be necessary. One could envisage an extrusion step, which would create the desired meat-like texture and, at the same time, degrade heat-labile toxins and denature toxic proteins.

## 11. The Potential of Fermenter Produced Fungal Mycelia as Future Meat Substitutes

Recent years have seen a flood of more or less dubious, but fashionable, nutraceuticals to combat supposed deficiencies in the diet. The real challenge of the coming years, however, will be to feed a growing global population. Both water and an ample supply of protein will increasingly become major issues facing coming generations. Basidiomycota, such as the above discussed edible fungi, occupy an intermediate taxonomic position between less developed microorganisms and the higher plants. Equipped with a complex biochemistry like plant cells, they grow much faster than in vitro plant cells and accept very different substrates without requiring expensive growth regulators. Fermenter cultivated mycelia accumulate protein with a higher biological value compared with prokaryotes, yeasts, and even plant sources, and they accumulate protein faster and with a smaller carbon dioxide footprint compared with macroalgae, insects, or plants.

A very recent study calculated that if only 20% of the beef consumed were replaced by fungal protein by 2050, the worldwide deforestation would be halved [97]. Less beef consumption means less pasture is required for grazing cattle, and fewer cattle would reduce the need for arable land to produce feed (or alternative plant proteins), which in turn would lead to less deforestation. Methane emission from rumination and nitrous oxide emissions from fertilisers and manure would also be reduced.

Fungal bioprocesses have the major advantage of being decoupled from agricultural production, can be operated anytime, anywhere and on varying scales according to demand. Originally developed from traditional food biotechnologies such as beer brewing or wine making, techniques of submerged cultivation of microorganisms have been used for decades for the production of organic and amino acids and other valuable compounds. To establish a circular bioeconomy, the water required by a fungal bioprocess may be purified and reused, nutrients may be supplied by renewable side-streams of food processing (Table 1), and energy may be delivered by solar panels or green hydrogen. The ultimate goal will be to provide consumers with a protein (or protein-rich product) indistinguishable in sensory and nutrient properties from pork or beef. The popularity of edible fungi should facilitate social acceptance of mycelial products. Thus, McCartney’s statement [5] may be expanded to “Less meat—less heat—but enjoyment without regrets”.

## 12. Differences between Fermenter Produced Fungal Mycelia and Fruiting Bodies

Differential gene expression in mycelia and fruiting bodies, which even continues during the aging of fruiting bodies [12], is simply a result of the exposure of the cells to different chemical and physical surroundings [19]. For example, presumed defence compounds, such as lectins [13], C8-volatiles [45] and agaritin [93], show higher concentrations in fruiting bodies because they are threatened by many kinds of predators (including humans). Although the total protein concentration and biological value of mycelia and fruiting bodies are similar [22,27], the monosaccharide building blocks, concentration and structure of polysaccharides are different. Nonetheless, all of the sources have been recommended “as immunomodulatory agents and potential immunotherapeutic medicines for patients with inadequate immune function” [37,41]. For medical applications, sterile grown mycelia appear to be more advantageous. 

The concentration of the membrane stiffening ergosterol was higher in mycelia [16,42], so illumination with UV-B would open a simple way to generate vitamin D_2_ enriched food supplements [98]. Concentrations of antioxidants are lower in mycelia, which corresponds with lower concentrations of polyunsaturated fatty acids and the different hypogeal oxygen and temperature levels [43,46]. In contrast, the concentration of lovastatin is higher in mycelia [44,69], perhaps because, unlike fruiting bodies, they have no need to emanate terpenoid allelochemicals. Levels of toxic metal ions including Cd, Pb and Hg are typically higher in fruiting bodies [43,50,71], and the large surface area of mycelial cells allows the efficient up-take of essential and therapeutic ions (“biofortification”), such as Zn, Fe [50] and Li [53].

A valid comparison of the chemical similarity of mycelia and fruiting bodies to assess “substantial equivalence” would require a “reference fruiting body” of known and constant composition. However, xylophilic saprophytes such as *A. bisporus*, *P. ostreatus* and *L. edodes* are characterised by a high adaptability to their habitat. They thrive on most of the substrates shown in Table 1 by quickly inducing large enzymatic networks (own experiments, data not shown). As a result, the composition of the fruiting bodies depends on the growth substrate [33,55,57,60,67,72,74], the strain chosen [33,60,74,75,76,77,78,80], its developmental stage [29,30,31,32,38], and the associated adverse or beneficial microbial community [83,84,85,86,87,88,89,90,91] and is thus variable like no other food. The same applies to the mass production of fungal mycelia. Parameters of fungal bioprocesses can be easily manipulated, and the often-dramatic metabolic changes monitored either on-line or by off-line sampling [60,72]. A prototypic study on *P. eryngii* varied temperature, pH, energy input, carbon sources, nitrogen sources, and the C/N ratio in shake flasks and transferred the results to a 100 L batch cultivation [99]. The mycelial biomass incorporated enhanced levels of micronutrients from the nutrient medium, and UV-B illumination lifted the vitamin D_2_ concentration from 4.53 to 320 μg/g dry mass. 

This leads to the question of whether fermenter grown mycelia of edible fungi, if grown under food-grade conditions, should be legally classified as “novel foods”. Considering both the highly flexible biochemistry of mycelia and fruiting bodies and the lack of a prototypic fruiting body chemistry, it may be derived from the above comparisons that the application of the novel food regulation (EU) 2015/2283 encounters borders marked out by the nature of these peculiar foods. The appendix of (EU) 2015/2283 approved a “chitin-glucan” from the ascomycete *Aspergillus niger* and the basidiomycete *Fomes fomentarius*, a “chitosan extract” from *A. bisporus* and *A. niger* without specifying mycelia or fruiting bodies as a source. Perhaps implicitly considering the above outlined problems, a legal differentiation was not carried out. Among the fungi mentioned, only *A. bisporus* is edible. Strains of *A. niger* produce organic acids and enzymes in classical bioprocesses, but there are also mycotoxin forming strains known (ochratoxin, koji acid). *Fomes fomentarius* (touchwood) is feared as dry rot that destroys entire houses. 

Finally, commercially available mushrooms may be contaminated with microorganisms, insect eggs, agro-chemicals and heavy metals, even if properly produced. Wild mushrooms suffer more severely from such problems. The German *Bundesamt für Strahlenschutz* has pointed out that increased concentrations of Cs-137 are still present in some regions affected by the Chernobyl fallout of 1986 [100]. All of these health risks are principally excluded by advanced fungal biotechnology.

## 13. Conclusions

The quality and safety of foods is subject to legal regulations set by the responsible authorities worldwide. They will question if fermenter grown mycelia of edible fungi are chemically safe if they are worse or better than the fruiting bodies we have long consumed. Questions remain about how to prevent consumers being misled and whether mycelia differ from fruiting bodies to such an extent that their expected consumption would be nutritionally disadvantageous. Answers can only be based on a scientific evaluation of the composition, nutritional value, metabolism, and the level of (un)desirable constituents.

The data discussed above suggest that there is little scientifically founded reason to doubt the safety and nutritional value of mycelia from edible fungi if they are produced under controlled conditions in a defined environment. The production of citric acid by *A. niger* may be cited, which is regarded safe, after it was shown that mycotoxin formation is suppressed under industrial production conditions. This must be proven analogously for any industrially produced mycelia from basidiomycetes (due diligence of the producer, for example article 3 No. 3 (EU) 178/2002). Appropriate labelling in the list of ingredients of a food item will be mandatory. The risk of consuming undesired or toxic constituents in mycelia of edible basidiomycetes appears to be much lower than in consuming ascomycete sources such as Quorn.

## Figures and Tables

**Table 1:** Agro-industrial side-streams added successfully to submerged grown mycelia of Basidiomycota.

Side Stream	Reference(s)
Remainders from the production of fruiting bodies	[54]
Straw from cereals and leguminous plants	[55,56]
Sugar beet fibre	(own experiments, data not shown)
Bran from wheat, corn, rice	[57,58,59]
Peel from potato, apple, grape, citrus, banana	[23,60,61,62,63]
Cabbage cuttings	[64]
Press cake from oil production	[65]
Empty sunflower blossoms, peanut shells	[57,59,66]
Corn cobs	[55,56,67]
Draff and pomace from beer and winemaking	[62,68]
Sugar beet bagasse	[58]

## References

[1] United Nations (2015). We Can End Poverty—Millenium Development Goals and Beyond. https://www.un.org/millenniumgoals/.

[2] European Commission Cluster 6: Food, Bioeconomy, Natural Resources, Agriculture and Environment. https://ec.europa.eu/info/research-and-innovation/funding/funding-opportunities/funding-programmes-and-open-calls/horizon-europe/cluster-6-food-bioeconomy-natural-resources-agriculture-and-environment_en.

[3] European Commission Europäischer Grüner Deal. https://ec.europa.eu/info/strategy/priorities-2019-2024/european-green-deal_de.

[4] Xu X., Sharma P., Shu S., Lin T.-S., Ciais P., Tubiello F.N., Smith P., Campbell N., Jain A.K. (2021). Global greenhouse gas emissions from animal-based foods are twice those of plant-based foods. Nat. Food.

[5] McCartney P. (2019). Less Meat, Less Heat.

[6] Palanisamy M., Franke K., Berger R.G., Heinz V., Topfl S. (2019). High moisture extrusion of lupin protein: Influence of extrusion parameters on extruder responses and product properties. J. Sci. Food Agric..

[7] Kurtzman R.H. (1978). Production of Mushroom Fruiting Bodies on the Surface of Submerged Cultures. Mycologia.

[8] Sailaja B., Radhika B. (2018). A review on production of edible mushrooms and their applications. Int. J. Pharm. Biol. Sci..

[9] Burger F., Koch M., Fraatz M.A., Omarini A.B., Berger R.G., Zorn H. (2022). Production of an Anise- and Woodruff-like Aroma by Monokaryotic Strains of *Pleurotus sapidus* Grown on Citrus Side Streams. Molecules.

[10] Beelman R.B., Royse D.J., Chikthimmah N. (2003). Bioactive Components in Button Mushroom *Agaricus bisporus* (J. Lge) Imbach (Agaricomycetideae) of Nutritional, Medicinal, and Biological Importance. Int. J. Med. Mushrooms.

[11] Song H.-Y., Kim D.-H., Kim J.-M. (2018). Comparative transcriptome analysis of dikaryotic mycelia and mature fruiting bodies in the edible mushroom *Lentinula edodes*. Sci. Rep..

[12] Wang Y., Zeng X., Liu W. (2018). De novo transcriptomic analysis during *Lentinula edodes* fruiting body growth. Gene.

[13] Vetchinkina E., Fomin A., Navolokin N., Shirokov A. (2022). Proteins and polysaccharides from vegetative mycelium of medicinal basidiomycete *Lentinus edodes* display cytotoxicity towards human and animal cancer cell lines. Int. J. Biol. Macromol..

[14] Gao S., Wang G., Huang Z., Lei X., Bian Y., Liu Y., Huang W. (2018). Selection of Reference Genes for qRT-PCR Analysis in *Lentinula edodes* after Hot-Air Drying. Molecules.

[15] Lu Y.P., Liao J.H., Guo Z.J., Cai Z.X., Chen M.Y. (2020). Genome Survey and Transcriptome Analysis on Mycelia and Primordia of *Agaricus blazei*. Biomed. Res. Int..

[16] Fan X.-Z., Yao F., Yin C.-M., Sihi D.-F., Gao H. (2021). Exogenous Induction of Ergosterol Synthesis in *Agaricus blazei*. Mod. Food Sci. Technol..

[17] De Groot P.W., Schaap P.J., Sonnenberg A.S., Visser J., Van Griensven L.J. (1996). The *Agaricus bisporus* hypA gene encodes a hydrophobin and specifically accumulates in peel tissue of mushroom caps during fruit body development. J. Mol. Biol..

[18] Xu D., Wang Y., Keerio A.A., Ma A. (2021). Identification of hydrophobin genes and their physiological functions related to growth and development in *Pleurotus ostreatus*. Microbiol. Res..

[19] Qi Y., Sun X., Ma L., Wen Q., Qiu L., Shen J. (2020). Identification of two *Pleurotus ostreatus* blue light receptor genes (PoWC-1 and PoWC-2) and in vivo confirmation of complex PoWC-12 formation through yeast two hybrid system. Fungal Biol..

[20] Ye D., Du F., Zou Y., Hu Q. (2021). Transcriptomics Analysis of Primordium Formation in *Pleurotus eryngii*. Genes.

[21] Belitz H.-D., Grosch W., Schieberle P. (2004). Food Chemistry.

[22] Zajul M.M. (2017). Biotechnologische Produktion von Basidiomyceten-Proteinen auf industriellen Nebenströmen zur Herstellung von Nahrungsmitteln. Ph.D. Thesis.

[23] Ahlborn J., Stephan A., Meckel T., Maheshwari G., Rühl M., Zorn H. (2019). Upcycling of food industry side streams by basidiomycetes for production of a vegan protein source. Int. J. Recycl. Org. Waste Agric..

[24] Onuoha S.C., Okoroh P.N., Tom-Quinn R.A. (2021). Proximate Composition, Essential Heavy Metal Concentrations and Nutrient Density of the Mycelium and Fruiting Bodies of Organically Cultivated *Pleurotus ostreatus*. J. Appl. Life Sci. Int..

[25] Vetter J. (1995). [Mineral and amino acid contents of edible, cultivated shii-take mushrooms (*Lentinus edodes*)]. Z. Lebensm. Unters. Forsch..

[26] Valenzuela-Cobos J.D., Grijalva-Endara A., Marcillo-Vellejo R., Garcés-Moncayo M.F. (2020). Production and characterization of reconstituted strains of *Pleurotus* spp. cultivated on different agricultural wastes. Rev. Mex. De Ing. Química.

[27] Wang Y., Xia W., Li Z., Huang L., Ban L., Peng Y. (2017). Comparison and analysis of amino acid of fruit body and mycelium in *Agaricus brunnescens* Peck. Food Res. Dev..

[28] Finnigan T.J.A., Wall B.T., Wilde P.J., Stephens F.B., Taylor S.L., Freedman M.R. (2019). Mycoprotein: The Future of Nutritious Nonmeat Protein, a Symposium Review. Curr. Dev. Nutr..

[29] Wood D.A., Goodenough P.W. (1977). Fruiting of *Agaricus bisporus*. Arch. Microbiol..

[30] Ding Q., Zhao H., Zhu P., Jiang X., Nie F., Li G. (2022). Genome-wide identification and expression analyses of C2H2 zinc finger transcription factors in *Pleurotus ostreatus*. PeerJ.

[31] Xu D., Lu J., Wang Y., Keerio A.A., Zheng L., Chen L., Ma A. (2019). Identification and In Silico Analysis of Lectins in Gray Oyster Culinary-Medicinal Mushroom *Pleurotus ostreatus* (Agaricomycetes) Based on the Transcriptomes. Int. J. Med. Mushrooms.

[32] Sakamoto Y., Watanabe H., Nagai M., Nakade K., Takahashi M., Sato T. (2006). Lentinula edodes tlg1 encodes a thaumatin-like protein that is involved in lentinan degradation and fruiting body senescence. Plant Physiol..

[33] Manzi P., Gambelli L., Marconi S., Vivanti V., Pizzoferrato L. (1999). Nutrients in edible mushrooms: An inter-species comparative study. Food Chem..

[34] Zhang Y., Venkitasamy C., Pan Z., Liu W., Zhao L. (2017). Novel Umami Ingredients: Umami Peptides and Their Taste. J. Food Sci..

[35] Yamaguchi S., Yoshikawa T., Ikeda S., Ninomiya T. (1971). Measurement of the relative taste intensity of some L-α-amino acids 5′-nucleotides. J. Food Sci..

[36] Calonje M., García Mendoza C., Novaes-Ledieu M. (1996). New contributions to the wall polysaccharide structure of vegetative mycelium and fruit body cell walls of *Agaricus bisporus*. Microbiologia.

[37] Liu Y., Zheng D., Wang D., Su L., Wang Q., Li Y. (2019). Immunomodulatory Activities of Polysaccharides from White Button Mushroom, *Agaricus bisporus* (Agaricomycetes), Fruiting Bodies and Cultured Mycelia in Healthy and Immunosuppressed Mice. Int. J. Med. Mushrooms.

[38] Chen J., Li Q.-Z., Zhang L.-J., Song C.-Y., Shang X.-D., Gu Y.-F. (2020). Physico-chemical properties and immunocompetence in vitro of polysaccharides of *Lentinula edodes* fruiting bodies at different growth stages. Mycosystema.

[39] Sheng K., Wang C., Chen B., Kang M., Wang M., Liu K., Wang M. (2021). Recent advances in polysaccharides from Lentinus edodes (Berk.): Isolation, structures and bioactivities. Food Chem..

[40] Devi K.S.P., Behera B., Mishra D., Maiti T.K. (2015). Immune augmentation and Dalton’s Lymphoma tumor inhibition by glucans/glycans isolated from the mycelia and fruit body of *Pleurotus ostreatus*. Int. Immunopharmacol..

[41] Zheng X., Sun H., Wu L., Kong X., Song Q., Zhu Z. (2020). Structural characterization and inhibition on α-glucosidase of the polysaccharides from fruiting bodies and mycelia of *Pleurotus eryngii*. Int. J. Biol. Macromol..

[42] Cardoso R.V.C., Fernandes Â., Oliveira M., Calhelha R.C., Barros L., Martins A., Ferreira I. (2017). Development of nutraceutical formulations based on the mycelium of *Pleurotus ostreatus* and *Agaricus bisporus*. Food Funct..

[43] Ghahremani-Majd H., Dashti F. (2015). Chemical composition and antioxidant properties of cultivated button mushrooms (*Agaricus bisporus*). Hortic. Environ. Biotechnol..

[44] Kała K., Kryczyk-Poprawa A., Rzewińska A., Muszyńska B. (2020). Fruiting bodies of selected edible mushrooms as a potential source of lovastatin. Eur. Food Res. Technol..

[45] Kabbaj W., Breheret S., Guimberteau J., Talou T., Olivier J.M., Bensoussan M., Sobal M., Roussos S. (2002). Comparison of volatile compound production in fruit body and in mycelium of *Pleurotus ostreatus* identified by submerged and solid-state cultures. Appl. Biochem. Biotechnol..

[46] Papaspyridi L., Sinanoglou V., Strati I., Katapodis P., Christakopoulos P. (2013). Fatty acid profile of *Pleurotus ostreatus* and *Ganoderma australe* grown naturally and in a batch bioreactor. Acta Aliment..

[47] Tan Y.H., Moore D. (1994). High concentrations of mannitol in the shiitake mushroom Lentinula edodes. Microbios.

[48] Grembecka M. (2015). Sugar alcohols—their role in the modern world of sweeteners: A review. Eur. Food Res. Technol..

[49] Kokkoris V., Massas I., Polemis E., Koutrotsios G., Zervakis G.I. (2019). Accumulation of heavy metals by wild edible mushrooms with respect to soil substrates in the Athens metropolitan area (Greece). Sci. Total Environ..

[50] Muszyńska B., Krakowska A., Sułkowska-Ziaja K., Opoka W., Reczyński W., Baś B. (2015). In vitro cultures and fruiting bodies of culinary-medicinal *Agaricus bisporus* (white button mushroom) as a source of selected biologically-active elements. J. Food Sci. Technol..

[51] Zięba P., Kała K., Włodarczyk A., Szewczyk A., Kunicki E., Sękara A., Muszyńska B. (2020). Selenium and Zinc Biofortification of *Pleurotus eryngii* Mycelium and Fruiting Bodies as a Tool for Controlling Their Biological Activity. Molecules.

[52] Muszyńska B., Kała K., Włodarczyk A., Krakowska A., Ostachowicz B., Gdula-Argasińska J., Suchocki P. (2020). Lentinula edodes as a Source of Bioelements Released into Artificial Digestive Juices and Potential Anti-inflammatory Material. Biol. Trace Elem. Res..

[53] Falandysz J., Fernandes A.R., Meloni D. (2022). An overview of the lithium content and lithiation of the cultivable macrofungal species, *Agaricus bisporus* and *Pleurotus* spp.. Trends Food Sci. Technol..

[54] Rodríguez Pérez S., García Oduardo N., Bermúdez Savón R.C., Fernández Boizán M., Augur C. (2008). Decolourisation of mushroom farm wastewater by *Pleurotus ostreatus*. Biodegradation.

[55] Wang J.T., Wang Q., Han J.R. (2013). Yield, polysaccharides content and antioxidant properties of the mushroom *Agaricus* subrufescens produced on different substrates based on selected agricultural wastes. Sci. Hortic..

[56] Xu S., Wang F., Fu Y., Li D., Sun X., Li C., Song B., Li Y. (2020). Effects of mixed agro-residues (corn crop waste) on lignin-degrading enzyme activities, growth, and quality of Lentinula edodes. RSC Adv..

[57] Vlasenko E., Kuznetsova O. (2020). The Influence of Complex Additives on the Synthesis of Aroma Substances by Gray Oyster Culinary-Medicinal Mushroom, *Pleurotus ostreatus* (Agaricomycetes) during the Substrate Cultivation. Int. J. Med. Mushrooms.

[58] Li W., Chen W., Yang Y., Zhang J., Feng J., Yu H., Zhou S., Li X., Liu Y. (2017). Effects of culture substrates on taste component content and taste quality of Lentinula edodes. Int. J. Food Sci. Technol..

[59] Pinela J., Omarini A.B., Stojković D., Barros L., Postemsky P.D., Calhelha R.C., Breccia J., Fernández-Lahore M., Soković M., Ferreira I. (2020). Biotransformation of rice and sunflower side-streams by dikaryotic and monokaryotic strains of *Pleurotus sapidus*: Impact on phenolic profiles and bioactive properties. Food Res. Int..

[60] Krahe N.-K. (2021). Intraspezifische Variabilität Alkenspaltender Enzymaktivitäten in Monokaryotischen und Dikaryotischen Stämmen von *Pleurotus sapidus*. Ph.D. Thesis.

[61] Mshandete A.M., Mgonja J.R. (2009). Submerged liquid fermentation of some Tanzanian basidiomycetes for the production of mycelial biomass, exopolysaccharides and mycelium protein using wastes peels media. ARPN J. Agric. Biol. Sci..

[62] Papadaki A., Kachrimanidou V., Papanikolaou S., Philippoussis A., Diamantopoulou P. (2019). Upgrading Grape Pomace through *Pleurotus* spp. Cultivation for the Production of Enzymes and Fruiting Bodies. Microorganisms.

[63] Ding Z., Chen Y., Xu Z., Peng L., Xu G., Gu Z., Zhang L., Shi G., Zhang K. (2014). Production and characterization of laccase from *Pleurotus ferulae* in submerged fermentation. Ann. Microbiol..

[64] Grosse M., Wu S., Krings U., Berger R.G. (2020). Formation of Decatrienones with a Pineapple-like Aroma from 1-(13)C-Acetate by Cell Cultures of the Birch Polypore, Fomitopsis betulina. J. Agric. Food Chem..

[65] Ramachandran R., Jebapriya R., Gnanadoss J.J. (2013). Production of cellulase and laccase using *Pleurotus* sp. under submerged and solid-state fermentation. Int. J. Curr. Sci..

[66] Rebhun M., Hadar Y. (2005). Use of Agro-Industrial Waste for Production of Laccase and Manganese Peroxidase from White-Rot Basidiomycetes. Int. J. Med. Mushrooms.

[67] Win T.T., Ohga S. (2018). Study on the Cultivation of *Agaricus blazei* (Almond Mushroom) Grwon on Compost Mixed with Selected Agro-Residues. Adv. Microbiol..

[68] Wolters N., Schabronath C., Schembecker G., Merz J. (2016). Efficient conversion of pretreated brewer’s spent grain and wheat bran by submerged cultivation of *Hericium erinaceus*. Bioresour. Technol..

[69] Krakowska A., Zięba P., Włodarczyk A., Kała K., Sułkowska-Ziaja K., Bernaś E., Sękara A., Ostachowicz B., Muszyńska B. (2020). Selected edible medicinal mushrooms from *Pleurotus genus* as an answer for human civilization diseases. Food Chem..

[70] Malarczyk E., Zinko E., Nowak G., Ziaja J., Kochmanska-Rdest J., Leonowicz A. The relation between the concentration of phenolic activity of manganese peroxidase in cultures of two species of Pleurotus during fruit body formation. Proceedings of the TAPPI Biological Science Symposium.

[71] Siwulski M., Rzymski P., Budka A., Kalač P., Budzyńska S., Dawidowicz L., Hajduk E., Kozak L., Budzulak J., Sobieralski K. (2019). The effect of different substrates on the growth of six cultivated mushroom species and composition of macro and trace elements in their fruiting bodies. Eur. Food Res. Technol..

[72] Grosse M., Strauss E., Krings U., Berger R.G. (2019). Response of the sesquiterpene synthesis in submerged cultures of the Basidiomycete Tyromyces floriformis to the medium composition. Mycologia.

[73] Krahe N.-K., Berger R.G., Witt M., Zorn H., Omarini A.B., Ersoy F. (2021). Monokaryotic *Pleurotus sapidus* Strains with Intraspecific Variability of an Alkene Cleaving DyP-Type Peroxidase Activity as a Result of Gene Mutation and Differential Gene Expression. Int. J. Mol. Sci..

[74] Sarris D., Philippoussis A., Mallouchos A., Diamantopoulou P. (2020). Valorization of low-cost, carbon-rich substrates by edible ascomycetes and basidiomycetes grown on liquid cultures. FEMS Microbiol. Lett..

[75] Leong C.C., Ho W.Y., Yeap S.K., Krishnen G., Chong Z.X., Ho J.S., Lim P.T., Ten S.T. (2021). Assessment of phylogenetic, growth, and antioxidant capacity of *Pleurotus* spp. in Malaysia. J. Food Process. Preserv..

[76] Calabretti A., Mang S.M., Becce A., Castronuovo D., Cardone L., Candido V., Camele I. (2021). Comparison of bioactive substances content between commercial and wild-type isolates of *Pleurotus eryngii*. Sustainability.

[77] Zheng W.-M., Zhang L.-J., Li Q.-Z., Song C.-Y., Shang X.-D., Tan Q. (2021). Genetic law of important agronomic traits of *Lentinula edodes* in F2 and F3 generations. Mycosystema.

[78] Sousa M., Costa L., Malantrucco T., Pereira T., Bastos S., Dias E. (2016). Nutritional and enzymatic potential of the Lentinula edodes strains. Int. J. Curr. Res..

[79] Ryu S.-R., Bak W.-C., Koo C.-D., Lee B.-H. (2009). Studies on breeding and cultivation characteristics of *Lentinula edodes* strains for sawdust cultivation. Korean J. Mycol..

[80] Omarini A.B., Plagemann I., Schimanski S., Krings U., Berger R.G. (2014). Crosses between monokaryons of Pleurotus sapidus or *Pleurotus florida* show an improved biotransformation of (+)-valencene to (+)-nootkatone. Bioresour. Technol..

[81] Linke D., Omarini A.B., Takenberg M., Kelle S., Berger R.G. (2019). Long-Term Monokaryotic Cultures of *Pleurotus ostreatus* var. florida Produce High and Stable Laccase Activity Capable to Degrade ss-Carotene. Appl. Biochem. Biotechnol..

[82] Vreeburg S., Nygren K., Aanen D.K. (2016). Unholy marriages and eternal triangles: How competition in the mushroom life cycle can lead to genomic conflict. Philos. Trans. R. Soc. B.

[83] Dobbs E., Deakin G., Bennett J., Fleming-Archibald C., Jones I., Grogan H., Burton K. (2021). Viral interactions and pathogenesis during multiple viral infections in *Agaricus bisporus*. Mbio.

[84] Kumar B., Kumari C., Kumar M. (2018). Effect of bio-fertilizers on mycelial growth and physical properties of white button mushroom *Agaricus bisporus* (Lange) Imbach]. Int. J. Curr. Microbiol. App. Sci..

[85] Pandin C., Le Coq D., Deschamps J., Védie R., Rousseau T., Aymerich S., Briandet R. (2018). Complete genome sequence of Bacillus velezensis QST713: A biocontrol agent that protects *Agaricus bisporus* crops against the green mould disease. J. Biotechnol..

[86] Kertesz M.A., Thai M. (2018). Compost bacteria and fungi that influence growth and development of *Agaricus bisporus* and other commercial mushrooms. Appl. Microbiol. Biotechnol..

[87] Ghasemi S., Harighi B., Azizi A., Mojarrab M. (2020). Reduction of brown blotch disease and tyrosinase activity in *Agaricus bisporus* infected by *Pseudomonas tolaasii* upon treatment with endofungal bacteria. Physiol. Mol. Plant Pathol..

[88] Lakkireddy K., Khonsuntia W., Kües U. (2020). Mycoparasite Hypomyces odoratus infests *Agaricus xanthodermus* fruiting bodies in nature. AMB Express.

[89] Song H.-Y., Kim N., Kim D.-H., Kim J.-M. (2020). The PoV mycovirus affects extracellular enzyme expression and fruiting body yield in the oyster mushroom, *Pleurotus ostreatus*. Sci. Rep..

[90] Kumari S., Naraian R. (2021). Enhanced growth and yield of oyster mushroom by growth-promoting bacteria *Glutamicibacter arilaitensis* MRC119. J. Basic Microbiol..

[91] Bánfi R., Pohner Z., Szabó A., Herczeg G., Kovács G.M., Nagy A., Márialigeti K., Vajna B. (2021). Succession and potential role of bacterial communities during *Pleurotus ostreatus* production. FEMS Microbiol. Ecol..

[92] Liebenow H., Hahn A., Michalak H. (2005). Risiko Pilze—Einschätzung und Hinweise.

[93] Speroni J.J., Beelman R.B., Schisler L.C. (1983). Factors Influencing the Agaritine Content in Cultivated Mushrooms, *Agaricus bisporus*. J. Food Prot..

[94] Shephard S.E., Schlatter C. (1998). Covalent binding of agaritine to DNA in vivo. Food Chem. Toxicol..

[95] Sepcić K., Berne S., Potrich C., Turk T., Macek P., Menestrina G. (2003). Interaction of ostreolysin, a cytolytic protein from the edible mushroom *Pleurotus ostreatus*, with lipid membranes and modulation by lysophospholipids. Eur. J. Biochem..

[96] Stephany M.P., Chung S., Handler M.Z., Handler N.S., Handler G.A., Schwartz R.A. (2016). Shiitake Mushroom Dermatitis: A Review. Am. J. Clin. Dermatol..

[97] Humpenöder F., Bodirsky B.L., Weindl I., Lotze-Campen H., Linder T., Popp A. (2022). Projected environmental benefits of replacing beef with microbial protein. Nature.

[98] Krings U., Berger R.G. (2014). Dynamics of sterols and fatty acids during UV-B treatment of oyster mushroom. Food Chem..

[99] Singh U., Gautam A., Singha T.K., Tiwari A., Tiwari P., Sahai V., Sharma S. (2020). Mass production of *Pleurotus eryngii* mycelia under submerged culture conditions with improved minerals and vitamin D2. LWT.

[100] Bundesamt f. Strahlenschutz (2019). Wildpilze—Bedenkenloser Genuss?.

